# Supplementary material for: The persistent benefits of decreasing default pill counts for postoperative narcotic prescriptions
Source: PLoS One. 2024 Jun 4;19(6):e0304100. doi: 10.1371/journal.pone.0304100 (PMC11149874; doi:10.1371/journal.pone.0304100)
Supplement: S1 Table — Patient demographics as overall and broken down by year. (DOCX) [file pone.0304100.s001.docx]

S 1 Table. Patient demographics as overall and broken down by year.
