# Supplementary material for: The persistent benefits of decreasing default pill counts for postoperative narcotic prescriptions
Source: PLoS One. 2024 Jun 4;19(6):e0304100. doi: 10.1371/journal.pone.0304100 (PMC11149874; doi:10.1371/journal.pone.0304100)
Supplement: S2 Table — The raw count and percent of postoperative opioid prescriptions at the default MME, higher than the default MME, lower than the default MME, and resulting in refills from 2017–2021 based on prior opioid use or opioid naïve. (DOCX) [file pone.0304100.s002.docx]

| **Prior Opioid Use** | |  |  |  |
| --- | --- | --- | --- | --- |
| **Discharge Year** | **MME Pattern** | | | |
|  | **Default** | **High** | **Low** | **Total** |
| **2017** | 643 | 4847 | 592 | 6082 |
|  | 10.57% | 79.69% | 9.73% |  |
| **2018** | 1006 | 5585 | 879 | 7470 |
|  | 13.47% | 74.77% | 11.77% |  |
| **2019** | 1115 | 4614 | 1106 | 6835 |
|  | 16.31% | 67.51% | 16.18% |  |
| **2020** | 1061 | 4090 | 1216 | 6367 |
|  | 16.66% | 64.24% | 19.10% |  |
| **2021** | 1332 | 4604 | 1662 | 7598 |
|  | 17.53% | 60.59% | 21.87% |  |
| **Total** | 5157 | 23740 | 5455 | 34352 |

| **Naïve Opioid Use** | | | | |
| --- | --- | --- | --- | --- |
| **Discharge Year** | **MME Pattern** | | | |
|  | **Default** | **High** | **Low** | **Total** |
| **2017** | 4007 | 17383 | 5246 | 26636 |
|  | 15.04% | 65.26% | 19.7% |  |
| **2018** | 7665 | 20315 | 8684 | 36664 |
|  | 20.91% | 55.41% | 23.69% |  |
| **2019** | 8973 | 16207 | 9964 | 35144 |
|  | 25.53% | 46.12% | 28.35% |  |
| **2020** | 7388 | 11449 | 9061 | 27898 |
|  | 26.48% | 41.04% | 32.48% |  |
| **2021** | 7759 | 11331 | 11595 | 30685 |
|  | 25.29% | 36.93% | 37.79% |  |
| **Total** | 35792 | 76685 | 44550 | 157027 |

S2 Table. The raw count and percent of postoperative opioid prescriptions at the default MME, higher than the default MME, lower than the default MME, and resulting in refills from 2017-2021 based on prior opioid use or opioid naïve.
