# Supplementary material for: The persistent benefits of decreasing default pill counts for postoperative narcotic prescriptions
Source: PLoS One. 2024 Jun 4;19(6):e0304100. doi: 10.1371/journal.pone.0304100 (PMC11149874; doi:10.1371/journal.pone.0304100)
Supplement: S3 Table — The raw quantities of 90-day opioid refills and the 90-day refills as a percentage of total annual prescriptions based on prior opioid prescription or opioid-naïve patients. (DOCX) [file pone.0304100.s003.docx]

| **Prior Opioid Use** | | | |
| --- | --- | --- | --- |
| **Discharge Year** | **Refill** | | |
|  | **No** | **Yes** | **Total** |
| **2017** | 3848 | 2234 | 6082 |
|  | 63.27% | 36.73% |  |
| **2018** | 4676 | 2794 | 7470 |
|  | 62.60% | 37.40% |  |
| **2019** | 4347 | 2488 | 6835 |
|  | 63.60% | 36.40% |  |
| **2020** | 4039 | 2328 | 6367 |
|  | 63.44 | 36.56 |  |
| **2021** | 4686 | 2912 | 7598 |
|  | 61.67% | 38.33% |  |
| **Total** | 21596 | 12756 | 34352 |

| **Naïve Opioid Use** | | | | |  |
| --- | --- | --- | --- | --- | --- |
| **Discharge Year** | **Refill** |  |  |  | |
|  | **No** | **Yes** | **Total** |  | |
| **2017** | 22647 | 3989 | 26636 |  | |
|  | 85.02% | 14.98% |  |  | |
| **2018** | 31173 | 5491 | 36664 |  | |
|  | 85.02% | 14.98% |  |  | |
| **2019** | 29960 | 5184 | 35144 |  | |
|  | 85.25% | 14.75% |  |  | |
| **2020** | 23662 | 4236 | 27898 |  | |
|  | 84.82% | 15.18% |  |  | |
| **2021** | 25981 | 4704 | 30685 |  | |
|  | 84.67% | 15.33% |  |  | |
| **Total** | 133423 | 23604 | 157027 |  | |

S3 Table. The raw quantities of 90-day opioid refills and the 90-day refills as a percentage of total annual prescriptions based on prior opioid prescription or opioid-naïve patients.
